# Should Associations between HIV-Related Risk Perceptions and Behaviors or Intentions Be Positive or Negative?

**DOI:** 10.1371/journal.pone.0052124

**Published:** 2012-12-19

**Authors:** Hiyi Tsui, Joseph T. F. Lau, Weina Xiang, Jing Gu, Zixin Wang

**Affiliations:** 1 Centre for Health Behaviors Research, The Jockey Club School of Public Health and Primary Care, Faculty of Medicine, The Chinese University of Hong Kong, Hong Kong; 2 Centre for Medical Anthropology and Behavioral Health, Sun Yat-sen University, Guangzhou, China; 3 Department of Epidemiology, Rollins School of Public Health, Emory University, Atlanta, Georgia, United States of America; 4 School of Public Health, Sun Yat-sen University, Guangzhou, China; University of Ottawa, Canada

## Abstract

Risk perceptions are important in HIV research and interventions; mixed results were found between HIV-related perceptions and behaviors. We interviewed 377 sexually active injecting drug users in China, finding mixed associations between HIV-related risk perception assessed by two general measures and two previous risk behaviors (syringe sharing: p<.05; unprotected sex: p>.05) – partially supporting the ‘reflective hypothesis’ that reflection on previous behaviors increases risk perceptions. When we use specific measures for risk perceptions (HIV transmission via unprotected sex with specific types of sex partner and via syringe sharing) and use behavioral intention to adopt protective risk behaviors (condom use and avoid syringe sharing totally) as dependent variables, positive significant associations were observed – supporting the motivational hypothesis that risk perceptions motivate one to adopt protective behaviors. The direction and significance of the associations of concern depends on types of measures used. It has important implications on research design, data interpretation and services.

## Introduction

It is estimated that there are 780,000 HIV positive cases in China. In 2011, there were 92,940 reported HIV/AIDS cases of which 28.4% could be attributed to injection drug use [1], which has driven the HIV epidemic in China since 1989 [2], [3], [4]. The HIV prevalence among injection drug users (IDU) in China ranged from 6.4% to 71.9% [1], [5], [6]. A recent systematic review estimated that there are 2.9 million IDU in China [7]. Although it is commonly believed that some drugs (e.g. heroin) diminish sexual interest among IDU [8], [9], previous studies have revealed that many male IDU remain sexually active [10], [11], [12], [13], [14], [15]. IDU may engage in syringe sharing and unprotected sex and may hence face dual risks of HIV infection [16]. They form a potential ‘bridge’ transmitting HIV from the higher risk IDU population to the lower risk general population, such as their sex partners who are not IDU [17].

HIV-related risk perception is often measured by assessing one’s perceived likelihood of contracting HIV. It is a core element of prevailing health behavior theories [18] such as the Health Action Process Approach (HAPA) [19], [20] and the Health Belief Model [21], [22]. It is an important concept which can be applied to understand HIV-related behaviors and to develop HIV research and interventions. Theoretically, elevated risk perception of HIV infection should prompt individuals to adopt relevant precautionary measures and/or to refrain from adoption of risk behaviors. Attempts to raise awareness and risk perception are hence common strategies of HIV interventions [23], [24]. Risk perception is one of the most popular HIV-related research topics [25], [26], [27], [28], [29], [30], [31], [32]. Despite the significance, its relationship with risk behaviors has not been well studied, possibly due to conceptual and measurement issues.

Previous studies have reported mixed results of positive, negative and null associations between HIV-related risk perceptions and inconsistent condom use in the last few months [33], [34], [35], [36], intention to use condoms consistently in the future [37], [38], [39], previous behaviors of syringe sharing and syringe sharing in the future [40]. Such discrepancies challenge whether attempts to increase risk perception in HIV interventions are evidence-based. It is therefore important to look into methodological reasons beneath the inconsistencies.

One of the plausible reasons of the inconsistencies may be attributed to the cross-sectional study design being used in most of the aforementioned studies [41], [42], [43], [44], [45], [46], [47], which reported associations instead of causal relationships. Positive associations could be resulted if those who had practiced high risk behaviors (e.g. unprotected sex or syringe sharing) in the past would perceive higher chances of contracting HIV, as compared to those who did not practice high risk behaviors. This is known as the reflective hypothesis [48], which can operate in cross-sectional studies involving retrospectively assessed risk behaviors. In contrast, the motivational hypothesis [49] suggests that those who perceive higher chances in contracting HIV in the future would be motivated to adopt preventive behaviors (e.g. condom use or avoid syringe sharing) in the future in order to avoid contracting HIV, as compared to others. In cross-sectional studies, both mechanisms may operate simultaneously to affect the direction of the association, when the question asked involves previous risk behaviors and an unclear time frame with respect to risk perception (e.g. ‘How likely is it for you to contract HIV?’). The relative strength of the two mechanisms would determine the final direction of the association between HIV-related risk and previous behaviors.

As mixed results have also been observed in studies asked about intention to use condoms or intention to share syringes in the future [35], [36], [40] and those studies using longitudinal study design [50], other methodological problems, such as measurement issues, may co-exist with the aforementioned causality problem. The measures of HIV-related perceptions may or may not be specific to the nature of the risk behavior, such as types of risk behavior (e.g. condom use or syringe sharing) and people (e.g. type of sex partners) involved. The measurement of risk behaviors may either be general (e.g. condom use during sex with any sex partner) or specific (e.g. condoms use during sex with regular or non-regular sex partners). It is hypothesized that the use of general versus specific measures of risk perception and risk behaviors would yield associations of different directions.

The study investigated the prevalence of: 1) inconsistent condom use during sex with any female sex partners in the last six months (previous behavior), 2) intention to use condoms consistently during sex with three types of female sex partners (regular, non-regular and commercial female sex partners) in the next six months, 3) syringe sharing in the last six months (previous behavior), and 4) intention to avoid sharing syringes with others in the next six months totally.

HIV risk perception assessments included: 1) levels of two general unconditional sex-related HIV risk assessment variables (not conditional on any characteristics of risk behaviors), 2) one general drug-use-related HIV risk assessment, 3) three measures of sex-related HIV risk assessment conditional on unprotected sex respectively with three types of female sex partners (regular, non-regular and commercial female sex partners), and 4) one measure of drug-use-related HIV risk assessment conditional on syringe sharing with others (see Measures).

In this study, the reflection hypothesis was firstly tested by investigating the corresponding associations between two general HIV risk assessment variables and two types of previous HIV-related behaviors (i.e. inconsistent condom use with any female sex partner and syringe sharing with others in the last six months). It was hypothesized that those practicing such two types of previous risk behaviors would be more likely than others to reflect on the risk level caused by their previous risk behaviors and would therefore possess higher levels of general unconditional sex-related and unconditional drug-use-related HIV risk perceptions.

The motivational hypotheses were tested by investigating the corresponding associations between the four (three sex-related and one drug-use-related) specific HIV risk perceptions conditional on characteristics of the risk behaviors and the intention to avoid the corresponding risk behavior (condom use in the next six months or total avoidance of syringe sharing in the next six months). It was hypothesized that those with higher levels of specific HIV risk perceptions were more likely than others to be motivated to avoid risk behaviors in the future. They would hence have higher intention to use condoms consistently or to avoid syringe sharing totally in the next six months. The null hypotheses suggest statistically non-significant associations. This is one of the few studies tackling issues on the direction of the associations between HIV-related risk perceptions and risk behaviors and is the only one of the type conducted in China.

## Methods

### Ethics Statement

This study has been approved by the Research Survey Ethics Committee of the Chinese University of Hong Kong prior to commencement. Verbal informed consent from the participants was obtained before data collection. Participants were not asked to sign on the consent form as drug use was illegal in China. Instead, the interviewers signed a form pledging that they had explained the details of the study fully to the participants. This consent procedure has been approved by the ethics committees.

### Study Population and Sampling

A total of 429 male IDU were recruited from Dazhou city in Sichuan province, China during April through September, 2008. Inclusion criteria of this cross-sectional study were: 1) non-institutionalized male IDU, 2) being 18 to 45 years old, 3) having had injected drugs in the last six months, 4) self-reporting negative or unknown HIV status. Among the 429 IDU, 377 (87.9%) self-reported to be sexually active in the last six months. They were interviewed and their data were analyzed in this report.

### Data Collection Procedures

Participants were recruited via snowball sampling and outreach made by CDC staff. In addition, the sample included referrals made by peer educators of syringe exchange programs and users of HIV-related services. Prospective respondents were briefed about the study and were invited to participate in the study. Verbal informed consent was obtained prior to the commencement of the interview, using a structured questionnaire which took about 10 to 12 minutes to complete. Interviewers were staff of the local CDC and training was provided by the first author. Participants were not asked to sign on the consent form as drug use was illegal in China. Instead, the interviewers signed a form pledging that they had explained the details of the study fully to the participants. A small amount of money (RMB 20 to 30 = US$ 3 to 4.5) was given to the participants upon completion of the interview to compensate their time spent in the study. Similar procedures have been used in other published studies [14], [15]. Ethics approval was obtained from the Research Survey Ethics Committee of the Chinese University of Hong Kong.

### Measures

Background information was collected, including age, education level, drug use status, HIV-related knowledge and service utilization. HIV-related risk behaviors included multiple sex partnership, previous risk behaviors (inconsistent condom use with any female sex partner and syringe sharing with others) in the last six months. Questions further assessed intention to avoid practicing HIV-related risk behaviors (consistent condom use with regular sex partners (RP) or non-regular sex partners (NRP) or female sex workers (FSW), and intention to avoid syringe sharing totally in the next six months.

Two general (unconditional) HIV perception measures were used: perceived general absolute risk (PGAR) and perceived general relative risk (PGRR) – ‘How likely would you contract HIV?’ and ‘How likely would you contract HIV as compared to other peer IDU of your age?’ Three variables assessed specific sex-related HIV risk perception conditional on both unprotected sex and partner type: ‘How likely would you contract HIV if you have unprotected sex with regular sex partners?’ (PRCUS-RP) ‘How likely would you contract HIV if you have unprotected sex with non-regular sex partners?’ (PRCUS-NRP) ‘How likely would you contract HIV if you have unprotected sex with female sex workers?’ (PRCUS-FSW) Regular partners were defined as the participants’ spouse or girlfriends; non-regular partners were defined as those female sex partners who were not regular sex partners (e.g. one night stand) and did not involve exchanging money with sex; female sex workers were those sex partners involving exchange of money with sex. Another variable assessed HIV risk perception related to drug use conditional on syringe sharing with others (PRCSS): ‘How likely would you contract HIV if you share syringes with others?’.

### Statistical Analysis

Univariate odds ratios (OR) and respective 95% confidence intervals were derived to test the significance and strength of associations between the background variables and the six dependent variables on previous risk behaviors (last six months) and intentions to avoid risk behaviors (next six months). Multiple forward stepwise logistic regression models were fit, using all variables that were found to be significant in the univariate analysis as candidates for selection. To test the hypotheses, the associations between the risk perception measures (general and specific) and their corresponding dependent variable(s) on previous risk behaviors (sex-related and drug-use-related) and intention to avoid risk behaviors were tested by fitting multiple logistic regression models, adjusting for background variables that were found to be significant in the aforementioned forward stepwise logistic regression models. Statistical significance was set at 0.05 and SPSS 16.0 was used for data analysis.

## Results

### Background Characteristics

Of the participants, 71.9% were above 30 years old and 74.3% had not attended senior high schools. The majority of them had injected drugs for less than 10 years (78.8%) and had been doing so on a daily basis (68.2%), whilst 36.1% had had multiple female sex partners in the last six months. Only 25.3% of them had taken up voluntary HIV antibody counseling and testing (VCT) in the last six months, whilst 32.1% claimed that they had utilized more than three types of HIV prevention services (Table 1).

**Table 1:** Background characteristics of the participants (n = 377).

	%	(n)
Age group		
Below 30	28.1	(106)
30 and above	71.9	(271)
Education level		
≤Junior high	74.3	(280)
>Junior high	25.7	(97)
Knowledge on the asymptomatic property of HIV transmission^1^		
Inappropriate answer/uncertain	22.8	(86)
Appropriate answer	77.2	(291)
Duration of drug injection		
<10 years	78.8	(297)
≥10 years	21.2	(80)
Frequency of drug injection		
<once a day	31.8	(120)
At least once a day	68.2	(257)
Taken up HIV antibody testing in the last six months		
No	74.7	(281)
Yes	25.3	(95)
Number of HIV/STD-related services utilized (out of 5^2^) in the last six months		
None to three	67.9	(256)
Three to five	32.1	(121)
Multiple sex partners in the last six months		
No	63.9	(241)
Yes	36.1	(136)

1 A health-looking person infected with HIV could transmit HIV to others.

2 Including MMT, SEP, distribution of free condoms, STD checkup/treatment, and HIV/STD education materials.

### HIV-related Risk Behaviors and Intention to Avoid Risk Behaviors

All participants were sexually active in the last six months: 68.2% had had sex with RP; 25.7% had had sex with NRP, and 28.4% had had sex with FSW. Regarding previous risk behaviors adopted in the last six months, the majority of them (81.7%) were inconsistent condom users (with any female sex partners), whilst 10.1% had shared syringes with others. Regarding behavioral intention to avoid risk behaviors, respectively 36.2%, 50.5% and 63.6% intended to use condoms consistently with RP, NRP and FSW in the next six months, whilst the majority of them (82%) intended to avoid totally syringe sharing with others in the next six months (Table 2).

**Table 2:** Previous risk behaviors in the last six months, intention to avoid risk behaviors in the next six months, general and specific HIV-related risk perception measures.

	%	(n)
Previous behaviors in the last 6 months		
Inconsistent condom use with any female partner(s)		
No	18.3	(69)
Yes	81.7	(308)
Shared syringes with others		
No	89.9	(339)
Yes	10.1	(38)
Intention to avoid risk behaviors in the next 6 months		
Intention to use condoms consistently (every time) when having sex with RP#		
Lower chance/no chance	63.8	(164)
High/sure chance	36.2	(93)
Intention to use condoms consistently (every time) when having sex with NRP#		
Lower chance/no chance	49.5	(48)
High/sure chance	50.5	(49)
Intention to use condoms consistently (every time) when having sex with FSW#		
Lower chance/no chance	36.4	(39)
High/sure chance	63.6	(68)
Intention to avoid syringe sharing totally		
No	18.0	(68)
Yes	82.0	(309)
General unconditional risk perception measures		
Perceived general absolute risk [PGAR] (*How likely would you contract HIV?*)		
Low chance/no chance	87.5	(330)
High/sure chance of HIV infection	12.5	(47)
Perceived general relative risk [PGRR] *(How likely would you contract HIV, as compared to other peer male IDU of your age)*		
Low chance/no chance	93.9	(354)
Higher/much higher of HIV infection	6.1	(23)
Specific measures conditional on HIV-related risk behaviors		
Perceived risk conditional on unprotected sex with RP# [PRCUS(RP)] (*How likely would you contract HIV, if you did not use a condom when having sex with RP?*)		
Low chance/no chance	88.3	(227)
High/sure chance of HIV infection	11.7	(30)
Perceived risk conditional on unprotected sex with NRP# [PRCUS(NRP)] (*How likely would you contract HIV, if you did not use a condom when having sex with NRP?*)		
Low chance/no chance	64.9	(63)
High/sure chance of HIV infection	35.1	(34)
Perceived risk conditional on unprotected sex with FSW# [PRCUS(FSW)] (*How likely would you contract HIV, if you did not use a condom when having sex with FSW?*)		
Low chance/no chance	49.5	(53)
High/sure chance of HIV infection	50.5	(54)
Perceived risk conditional on syringe sharing [PRCSS] (*How likely would you contract HIV, if you shared syringes with others?*)		
Low chance/no chance	26.5	(100)
High/sure chance of HIV infection	73.5	(277)

RP: Regular partners; NRP: Non-regular partners; FSW: Female sex workers.

# Among those having the respective type of sex partner in the last 6 months (i.e. RP: n = 257; NRP: n = 97; FSW: n = 107).

Of all participants, respectively 12.5% and 6.1% perceived high risk of contracting HIV in absolute or relative sense (PGAR and PGRR; Table 2). Among those with the three particular types of sex partners, respectively 11.7%, 35.1% and 50.5% perceived high risk of contracting HIV specifically via their RP, NRP and FSW (Table 2). Among all participants, 73.5% perceived high risk of contracting HIV via syringe sharing with others (Table 2).

### Associations between Background Characteristics and HIV-related Behaviors/intention (with Previous Risk Behaviors in the Last Six Months and with Intention to Avoid Risk Behaviors in the Next Six Months)

In both the univariate and multivariate analyses, three background variables were significantly associated with inconsistently condom use with any female sex partners in the last six months: knowledge on the property of asymptomatic HIV transmission (multivariate OR = 0.44; 95% CI: 0.21, 0.94), duration of drug injection (multivariate OR = 2.65; 95% CI: 1.15, 6.10), and number of HIV/STD-related service utilized in the last six months (multivariate OR = 0.47; 95% CI: 0.27, 0.81). Multiple sex partnership in the last six months (OR = 4.54; 95% CI: 2.19, 9.27) was the only variable that was significantly associated with syringe sharing in the last six months in the univariate analysis.

The number of HIV/STD-related services utilized in the last six months was the only variable that was associated with intention to use condoms consistently with RP in the future six months (OR = 3.73; 95% CI: 2.16, 6.42). Similarly, knowledge about the asymptomatic property of HIV transmission was the only variable associated with intention to use condoms consistently with NRP (OR = 4.04; 95% CI: 1.04, 15.72) and with intention to use condoms consistently with FSW (OR = 2.92; 95% CI: 1.19, 7.15) in the next six months.

Three background factors were significantly associated with intention to avoid syringe sharing in the next six months totally, both in the univariate and multivariate analysis: knowledge on the asymptomatic property of HIV transmission (multivariate OR = 2.23; 95% CI: 1.25, 3.99), number of HIV/STD-related service utilized in the last six months (multivariate OR = 2.03; 95% CI: 1.07, 3.85), and multiple sex partnership in the last six months (multivariate OR = 0.54; 95% CI: 0.31, 0.92). The aforementioned background variables that were found to be significant in the multivariate analyses (data not tabulated) were adjusted for in subsequent data analysis.

### Adjusted Associations between General Risk Perception Measures and Previous Risk Behaviors – Testing the ‘Reflective Hypotheses’

Adjusting for the corresponding significant background variables, the two general (unconditional) HIV risk perception measures (PGAR and PGRR) were not statistically associated with the general measure of the previous experience of inconsistent condom use with any female sex partner in the last six months (Table 3). However, similar adjusted analysis showed that the two general (unconditional) HIV risk perception measures were significantly associated with previous experience of syringe sharing in the last six months (PGAR: AOR = 4.69, 95% CI = 2.15, 10.24; PGRR: AOR = 5.92, 95% CI = 2.19, 16.03; Table 4). The reflective hypothesis was hence supported in the case of syringe sharing but not in the case of condom use.

**Table 3:** Associations between general and specific measures of HIV risk perception & condom use previous behaviors and future intentions.

	Inconsistent condom use withany female partner(last 6 months)			Intended consistent condom use(next 6 months)								
Perceived chance of HIV infection				with RP#			with NRP#			with FSW#		
	Row%	OR_u_(95% CI)	OR_adj_(95% CI)	Row%	OR_u_(95% CI)	OR_adj_(95% CI)	Row%	OR_u_(95% CI)	OR_adj_(95% CI)	Row%	OR_u_(95% CI)	OR_adj_(95% CI)
General measures												
PGAR												
Low./no chance	82.1	1.00	1.00									
High/sure chance	78.7	0.81(0.38, 1.71)	0.96(0.44, 2.11)									
PGRR												
Low/no chance	80.8	1.00	1.00									
Higher/much higher	95.7	5.23(0.69, 39.49)	3.98(0.52, 30.76)									
Specific measures												
PRCUS(RP)#												
Low/no chance	NA	NA	NA	33.0	1.00	1.00	NA	NA	NA	NA	NA	NA
High/sure chance				60.0	3.04**(1.39, 6.64)	3.70**(1.62, 8.45)						
PRCUS(NRP)#												
Low/no chance	NA	NA	NA	NA	NA	NA	41.3	1.00	1.00	NA	NA	NA
High/sure chance							67.6	2.98*(1.24, 7.15)	3.04*(1.23, 7.49)			
PRCUS(FSW)#												
Low/no chance	NA	NA	NA	NA	NA	NA	NA	NA	NA	41.5	1.00	1.00
High/sure chance										85.2	8.10***(3.20, 20.51)	7.65***(2.98, 19.63)

# Among those having the respective type of sex partner in the last 6 months (i.e. RP: n = 257; NRP: n = 97; FSW: n = 107).

PGAR: Perceived general absolute risk.

PGRR: Perceived general relative risk.

PRCUS(RP): Perceived risk conditional on unprotected sex with RP; PRCUS(NRP): Perceived risk conditional on unprotected sex with NRP; PRCUS(FSW): Perceived risk conditional on unprotected sex with FSW.

OR_u_: Univariate odds ratio.

OR_adj_: Odds ratio adjusted for significant background factor(s).

* p<0.05;

** p<0.01;

*** p<0.001.

NA: not applicable.

**Table 4:** Associations between general and specific measures of HIV risk perception and behaviors/intentions related to syringe sharing.

Perceived chance of HIV infection	Syringe-sharing with others(last 6 months)			Intended to avoid totally sharing syringes with others (next 6 months)		
	Row%	OR_u_(95% CI)	OR_adj_(95% CI)	Row%	OR_u_(95% CI)	OR_adj_(95% CI)
General measures						
PGAR						
Low./no chance	7.3	1.00	1.00			
High/sure chance	29.8	5.41***(2.55, 11.46)	4.69***(2.15, 10.24)			
PGRR						
Low/no chance						
Higher/much higher chance	8.5	1.00	1.00			
	34.8	5.76*** (2.26, 14.69)	5.92***(2.19, 16.03)			
Specific measures						
PRCSS						
Some chance	NA	NA	NA	72.0	1.00	1.00
No chance at all				85.6	2.30**(1.33, 4.00)	2.09*(1.18, 3.68)

PGAR: Perceived general absolute risk.

PGRR: Perceived general relative risk.

PRCSS: Perceived risk conditional on syringe sharing.

OR_u_: Univariate odds ratio.

OR_adj_: Odds ratio adjusted for significant background factor(s).

* p<0.05;

** p<0.01;

** p<0.001.

NA: Not applicable.

### Adjusted Associations between Specific Sex-related Risk Perception Measures and Intention to Avoid Risk Behaviors in the Next Six Months

Among those with RP, NRP and FSW, the three corresponding specific and conditional sex-related risk perception measures were all significantly associated with intention to use condoms consistently with the three corresponding types of female sex partners in the next six months: RP (PRCUS-RP: AOR = 3.70, 95% CI = 1.62,8.45), NRP (PRCUS-NRP: AOR = 3.04, 95% CI = 1.23,7.49) and FSW (PRCUS-FSW: AOR = 7.65, 95% CI = 2.98,19.63) (see Table 3). Similarly, adjusting for the significant background variables, the aforementioned specific measures on HIV risk perception were significantly associated with intention to avoid syringe with others in the future six months totally (PRCSS: AOR = 2.09, 95% CI = 1.18, 3.68; Table 4). The motivational hypotheses were hence supported by these findings basing on specific HIV risk perception measures.

## Discussion

Corroborating with the results of previous studies [51], the sampled male IDU were at high risk of sexual transmission of HIV/STD – about 90% of them were sexually active and the majority of whom (>80%) were inconsistent condom users. The claim that the majority of IDU would have their sexual drive suppressed was not supported by our data. The prevalence of syringe-sharing among our participants was however, lower than that of other previous similar studies [52] and was consistent with previous findings obtained from local behavioral surveillance studies (personal communication with local CDC staff). It may be due to the extensive intervention efforts in the study sites, or reporting bias, or other unknown reasons.

The aforementioned observations on a high level of sexual risk but relatively low prevalence of syringe sharing suggested that male IDU might have overlooked the risk of sexual transmission of HIV. They might feel that the risk of HIV transmission via sex would be much lower than that via syringe sharing. Our results support the contention. It is interesting to see that the overall or general risk perception in this study population was low (around 10% felt susceptible for contracting HIV). However, the level of risk perception conditioned on syringe sharing was very high and the levels of risk perceptions conditioned on various types of female sex partner varied and were in between those of general risk perception and syringe sharing. Moreover, whilst harm reduction services such as methadone maintenance treatment targeting IDU in China are commonly available and are relatively effective [53], [54], such interventions might have underemphasized prevention of sexual transmission of HIV. The results reminded us of the importance of condom use promotion in the study population. Integrated services are required to reduce dual risks of HIV transmission faced by IDU in China.

Our findings showed that general risk perception was not significantly associated with previous sexual risk behaviors (inconsistent condom use in the last six months), reinforcing the contention that sexual risk may have been under-emphasized. General risk perception was associated with previous syringe sharing behaviors, supporting the reflection hypothesis - previous risk behaviors might have led to increase in current HIV risk perceptions: ‘As I had had unprotected sex, my chance to contract HIV should be high’.

The results were totally different when behavior-and-partner-specific risk perceptions replaced general measures and behavioral intention on consistent condom use, as well as when behavioral intentions on avoiding syringe sharing totally replaced previous syringe sharing. Such conditional risk perceptions were consistently and strongly associated with behavioral intentions to avoid both sex-related and drug-related risk behaviors in the future. Instead of supporting the aforementioned reflection hypothesis, the results hereby supported the motivational hypothesis – a higher level of conditional risk perception motivates individuals to avoid risk behaviors in the future (‘As the chances of HIV transmission associated with unprotected sex and syringe sharing are high, I would avoid such risk behaviors in the future’).

The results of the aforementioned hypothesis testing have strong implications on research design and interpretation of research results. First, researchers should be reminded that the choice of different types of risk perception measures in HIV-related studies (general or specific) and the choice of time frame (previous behavior or future intention) regarding the measures would have direct and strong impacts onto the results. Cautions are hence required. Furthermore, our results have encouraging implications on the design of HIV prevention services. It is still important to remind male IDU of the high risks involved in drug-related and sex-related behaviors. According to our results, such interventions would increase their intention to adopt HIV-related protective measures in the future.

With the harm reduction programs such as MMT in place, health communication messages about HIV risk associated with syringe sharing seem to have been better received as compared to those associated with sexual risk among male IDU. The risk perception involving unprotected sex with female RP of IDU was especially low. Many female RP of male IDU are IDU and are exposed to high risk of HIV transmission via drug use or sex work [55]. Amongst male IDU, there might be a false sense of security regarding low risk of HIV sexual transmission via female RP.

This may be the only study comparing the relationships between various types of conditional/unconditional HIV-related risk perceptions and various types of risk behaviors/intentions (sex-related behaviors with different types of partners and drug-related behavior) among male IDU. Our novel findings contribute to the understanding of the puzzle regarding previous inconsistent findings. Risk perception is a construct of key health behavioral theories. We contend that the aforementioned discrepancy can be partially attributed to the choice of measures on risk perception (general versus specific perception) and risk behaviors (previous behaviors versus future intention). We infer that risk perception is a useful construct in designing HIV prevention among male IDU.

It is warranted to develop and to refine measurements of conditional HIV risk perception. Researchers should be made aware about the heterogeneity and limitations on the currently used measures. Further research and debates are warranted. Such methodological development is a required step to advance behavioral health theories involving risk perceptions to another level. Its importance is not limited to HIV research. Moreover, though our findings were obtained from male IDU, the results should also be applicable to other groups that are vulnerable to HIV prevention, such as men who have sex with men (MSM). Many MSM in China are having unprotected sex with females. It is interesting to investigate risk perception directed to transmitting HIV from MSM to their female sex partners and the associations between such risk perceptions and condom use with female sex partners.

The study has some limitations. It only included male but not female IDU due to resource limitation. Many female IDU in China are FSW [56] who may possess different HIV-related risk perceptions and associations between such risk perceptions and behaviors or intentions. However, the issues discussed are general concerns in understanding other types of health behaviors in other populations. Another limitation was that most of the measures were self-constructed, though references have been made to previous studies. Few studies compared these measures. Limited by the length of the questionnaire, many measures were single-item indicators. Future studies are required to validate new conditional specific risk perception scales. Moreover, non-probabilistic sampling was performed as there was no sampling frame. Respondent driven sampling could have improved representativeness of the findings though other studies have used our study design [57]. Reporting bias due to social desirability may have been affected the findings [58] though the study was anonymous and privacy was ensured in data collection. As this was a cross-sectional study, we could only compare the scenarios using dependent variables of previous behaviors versus variables on future intentions, but not prospectively with future behaviors. Behavioral intention, a construct of the Theory of Planned Behaviors, is one of the strongest predictor of actual future behaviors [59]. Finally, the sample size of participants having had had sex with female sex workers and non-regular sex partners were relatively small.

In conclusion, our results suggested that the discrepancy of the relationship between HIV risk perception and risk behaviors/intentions may be partially explained by the choice of general versus specific tools for risk perception assessments. Both the reflective hypothesis (e.g. positive associations between risk perception and risk behavior) and the motivational hypothesis (e.g. negative associations between risk perception and future behavioral intention) could be supported by the data obtained from the same study, depending on the choice of measures. The support of the motivational hypothesis involving specific measures and behavioral intentions lends a warranted evidence base to the HIV prevention strategy in elevating HIV-related risk perception. HIV workers should be made aware of these results, which have both important theoretical and service implications. Future longitudinal studies are warranted to construct better risk perception measures, allowing for clearer interpretations. Such risk perception is an important but under-emphasized research area which should go beyond investigating HIV transmission among IDU.
